# The Elements of Data Sharing

**DOI:** 10.1016/j.gpb.2020.04.001

**Published:** 2020-04-28

**Authors:** Zhang Zhang, Shuhui Song, Jun Yu, Wenming Zhao, Jingfa Xiao, Yiming Bao

**Affiliations:** 1 China National Center for Bioinformation, Beijing 100101, China; 2 National Genomics Data Center, Beijing Institute of Genomics, Chinese Academy of Sciences, Beijing 100101, China; 3 CAS Key Laboratory of Genome Sciences and Information, Beijing Institute of Genomics, Chinese Academy of Sciences, Beijing 100101, China; 4 University of Chinese Academy of Sciences, Beijing 100101, China

Data and their tailored characteristics are inheritable and long-lived, surpassing their analyzed results and conclusions regardless if they are produced by their generators or users. Aside from designing experiments for the new acquisition, scientific researchers always begin with a thorough synthesis of the existing data, especially those that have been demonstrated authentic and timely. This fact has to be particularly emphasized more than ever, as all aspects of our daily life and its measurable activities, for better and worse, are being generated and recorded to be part of the collection—known as the BIG DATA.

## Sharing data is vital for a community of shared future

Sharing data begins with building a willful and dedicated community who consents a shared future at a global scale. On the one hand, public emergencies, such as epidemics and pandemics caused by many emerging infectious diseases, especially the two-in-a-row coronaviruses, severe acute respiratory syndrome coronavirus (SARS-CoV) and SARS-CoV-2 [1], often necessitate data sharing to aid expedited translation of big data into knowledge and procedures to improve human health. On the other hand, we are now being, and increasingly so, armed and empowered by many data-generating engines and tools, including high-throughput sequencing technologies and high-performance computing platforms, as well as their collaborative products—large-scale genomic big data that are generated at exponentially growing rates; most of the data are being continuously produced, often supported by public funding [2], [3]. Clearly, data sharing becomes pivotal for many considerations and plans for action in public emergencies, since the outcomes from data-sharing are of essence in yielding a complete picture of emergency situation, accelerating scientific research and knowledge discovery, and promoting sensible and expeditious decision-making as well.

Unfortunately, existing practices surrounding data sharing are not effective in achieving maximum interests from our investments. Data sharing is hindered or slowed down by a lack of clear identification of supporting elements for its implementation. What constitutes ‘the elements of data sharing’ is, however, largely undefined. Therefore, clarifying and defining data-sharing elements would be of fundamental significance. Especially, when the world faces unprecedented global threats and encounters public emergency situations (*e.g.*, SARS-CoV-2 has spread around more than 200 countries/regions with 2,213,653 infected cases and 154,462 deaths as of 18 April 2020), we, as a community of shared future, need to specify vital elements of data sharing and establish rapid, open, and effective data release norms.

## Data sharing demands a data ecosystem

Making data shared for the public involves a series of activities that span the entire life cycle of data flow and that embody all relevant parties in terms of policies for data sharing and release (particularly for data from public-funded research), standards for data description and exchange, as well as databases for data management and access. All these relevant entities and processes together form a data-sharing ecosystem, in which data sharing is initiated by data providers and implemented in databases that play important roles in data management and provide data access for the public. Therefore, elements of data sharing should cover two major camps, one for data providers (including not only raw data generators, but also databases that provide data annotations and relationships [4]) and the other for data managers.

## Promptness, openness, and usefulness are of essence for data providers

**Figure 1:**
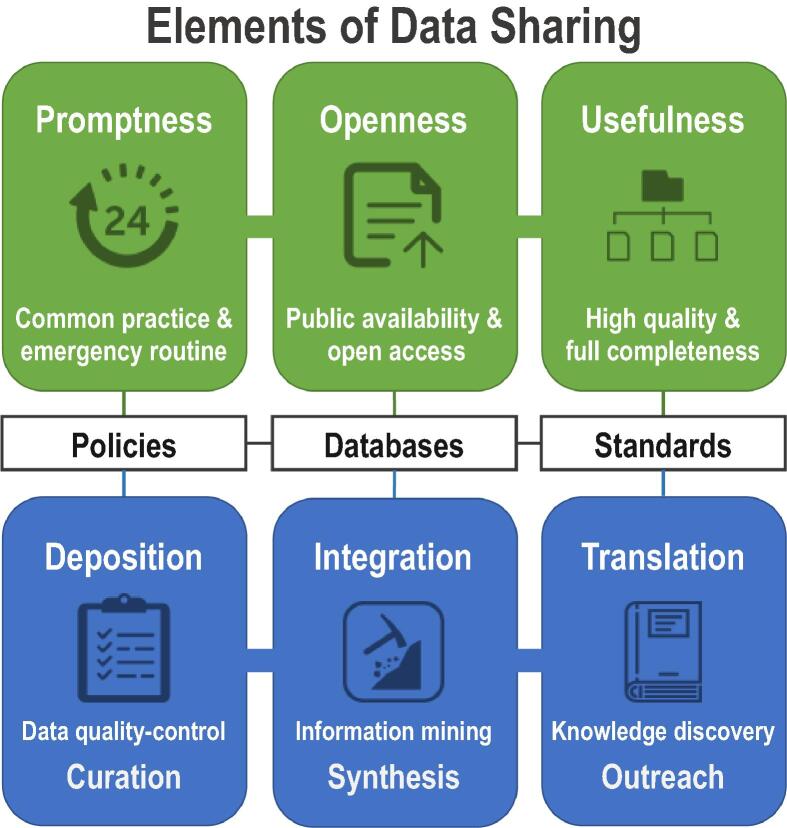
**The elements of data sharing** The elements of data sharing involve promptness, openness, and usefulness for data providers, as well as deposition, integration, and translation for data managers. In full support of data-sharing activities, policies, databases, and standards should be established and acknowledged by the whole scientific community.

For data providers, there are three key elements—*promptness*, *openness*, and *usefulness* (POU) —that serve as foundation guidelines for data sharing, particularly under public emergencies and critical situations (Figure 1). *Promptness* is crucially important during outbreaks since “*speed is everything*” [5]. It is consistent well with the Bermuda Principles, advocating rapid public release of genome sequence data within 24 h after generation and without restrictions on use proposed by the International Human Genome Sequencing Consortium in 1996. Given the unexpected emergency circumstances, sharing data in a timely manner is beneficial immediately for worldwide researchers and long-term for the global human society. Certainly, in this particular case, publication rights reserved for data providers is the major concern. In order to make both parties happy, policies for prompt data sharing as common practice and emergency routine are to be established, accepted, and monitored by the society, where detailed considerations and facts, such as criteria for intellectual property reservation, priority for publication, and credit for data providers [6], all must be thoroughly announced and debated in professional and public settings.

*Openness* emphasizes that both data themselves and the corresponding metadata should be released, publicized, and readily accessible in user-friendly databases. *“Nothing great is ever accomplished in isolation”*. Databases are not only responsible for data storage and processing, but also provide free internet access to all digital data. Currently, there have been several large global centers [7] in life sciences dedicated to molecular data (such as DNA/protein sequences and structures) collection and management, including the US National Center for Biotechnology Information (NCBI) [8], the European Bioinformatics Institute (EBI) [9], and the China National Center for Bioinformation/National Genomics Data Center (CNCB/NGDC) [10]. These publicly-supported centers accept data submissions globally and provide data-sharing services worldwide. It has to be emphasized that in order to keep data always accessible and long-lived, databases should be funded in a long-term and sustainable manner.

Last but not the least, the element of *usefulness* highlights the importance of data quality and completeness [11]. Data sharing is not a goal in itself but rather an effort to make data widely utilized. Accordingly, data to be shared must be reliable and complete, as biases/errors are characteristic of those in poor-quality or defective. Moreover, data in their full spectrum are definitely preferred, including all useful digital assets that contain, but not limited to, metadata, unprocessed data, derived datasets, analyzed results, source codes, protocols, flowcharts, *etc*. As a consequence, a collection of standards is certainly needed to be formulated by the user–provider community, and it can be envisaged that the more the community involvement is, the more successful the data-sharing efforts will become.

## Deposition, integration, and translation are of essence for data managers

In practice, data sharing in itself is only a single frame of its entire life cycle. In order to promote activities of data sharing, to provide easy access to all shared data, and to achieve full benefits from sharable data, databases must act as hub through providing a suite of web services for digital data *deposition*, *integration*, and *translation* (DIT) that are foundational elements for data management (Figure 1). After data submission, curation is conducted to certify the shared data with high quality and with the capability of reusability. Therefore, data curation involves a wide range of critical processes with standardized annotation, quality filtering, and value-added representation with controlled vocabularies. Only curated data can be used for further integration with the aim of information mining and synthesis processing. Consequently, translation of big data into knowledge discovery would be achieved, in company with various outreach activities for knowledge dissemination and application. After all, databases provide a core instrument for data management and coordinate the data-sharing ecosystem, orchestrating all important elements relevant to curation, synthesis, and outreach (Figure 1).

The POU–DIT Elements of data sharing are interrelated and can be used in any combination and evolve incrementally in response to the evolution of data ecosystems. They are applicable to a wide range of research fields, covering common aspects of data sharing in terms of timeliness, publicity, and content in POU, as well as data, information, and knowledge in DIT. Moreover, the POU–DIT Elements describing common conduct codes of data-sharing and guiding rules of data management are complementary to the FAIR Principles [12] (that define the characteristics of data, namely, Findable, Accessible, Interoperable, and Reusable). Obviously, they share common goals to promote data openness and reusability for the scientific community. Despite challenges in harmonizing with data ownership, security, privacy, and data-protection laws [2] (the European Union’s General Data Protection Regulation, the US Health Insurance Portability and Accountability Act, *etc.*), all important and complex issues would be best clarified via open discussions [13].

## Collaboration promotes data sharing

**Figure 2:**
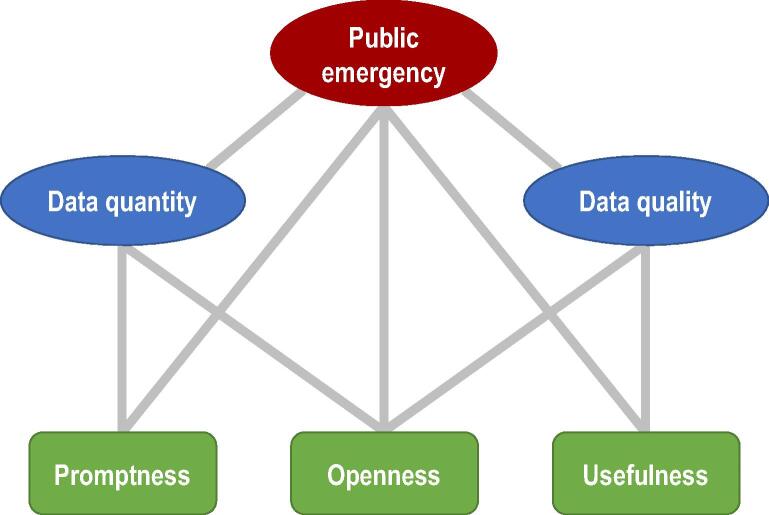
**Data-sharing scenarios in public emergency**

As mentioned above, challenges always come ahead of data sharing. For instance, diversity among data processing and sharing culture in a broadly-defined community, such as biomedicine—say genomics-meets-pandemics, often casts real obstacles. Ideally, funding agencies, journals, governmental organizations, as well as hands-on researchers, must work collaboratively and come up with common-practice protocols for data-sharing activities. Currently, a valuable effort is the Global Microbial Identifier (https://www.globalmicrobialidentifier.org) that aims to build a genomic epidemiological database for global identification of microorganisms in order to detect outbreaks and emerging pathogens. Ongoing efforts for the current outbreak caused by SARS-CoV-2 primarily include GISAID [14], GenBank [15] in NCBI, and the 2019 novel Coronavirus Resource [16] (2019nCoVR; https://bigd.big.ac.cn/ncov/) in CNCB/NGDC. Among them, 2019nCoVR features comprehensive integration and value-added curation, yielding large-quantity genome sequences with high-quality annotations (Figure 2) and providing a suite of services for viral genome data deposition, mining, and translation in real time. However, the need for data exchange and coordination between different databases, linking genomic data with important metadata, and data standardization across countries and laboratories, becomes very urgent and critical. To deal with global outbreaks as the COVID-19 pandemic, large and effective collaborations across different database resources (*e.g.*, 2019nCoVR, GISAID, and GenBank), disciplines, and countries towards data sharing are of immediate necessity.

## Data planet welcomes data sharing

Collectively, data sharing is vital for translating data to knowledge, particularly when everyone in the world faces the same threat. To maximize benefits of data sharing for everyone, the POU–DIT Elements must establish logistics and standards for data sharing, provide guidance for all users that include, but not limited to, scientific researchers, policy makers, funding agencies, and journal publishers, and carry out all data-sharing activities. Some of the data and related infrastructures built in the processes, aside from the immediate utilization, may form historic memoirs and monuments for both heroes and victims of the event. Nevertheless, we need to embrace a data-sharing culture under both ordinary and extraordinary situations [17]. With shared future, we call upon our professional colleagues to hold our hands together and collaborate full-heartedly to build a better data planet, where data produced by the global community are shared with the POU–DIT Elements.

## Competing interests

The authors declare no competing interests.
